# External validation of model predicting healthcare costs using cohort study-derived and claims-derived predictor variables in community-dwelling older adults

**DOI:** 10.1371/journal.pone.0354978

**Published:** 2026-08-12

**Authors:** Lisa Langsetmo, John T. Schousboe, Allyson M. Kats, Brent C. Taylor, Cynthia M. Boyd, Kerry M. Sheets, Kristine Ensrud

**Affiliations:** 1 Department of Medicine, University of Minnesota, Minneapolis, Minnesota, United States of America; 2 Division of Epidemiology & Community Health, School of Public Health, University of Minnesota, Minneapolis, Minnesota, United States of America; 3 Center for Care Delivery & Outcomes Research, VA Healthcare System, Minneapolis, Minnesota, United States of America; 4 HealthPartners Institute, Bloomington, Minnesota, United States of America; 5 Divison of Health Policy & Management, School of Public Health, University of Minnesota, Minneapolis, Minnesota, United States of America; 6 School of Medicine, Johns Hopkins University, Baltimore, Maryland, United States of America; 7 Department of Health Policy & Management, Johns Hopkins University, Baltimore, Maryland, United States of America; 8 Department of Epidemiology, Johns Hopkins University, Baltimore, Maryland, United States of America; 9 Department of Medicine, Hennepin Healthcare, Minneapolis, Minnesota, United States of America; Ehime University Graduate School of Medicine, JAPAN

## Abstract

**Background:**

In a pooled analysis of 4 prospective cohort studies linked with Medicare claims (2002–2011), claims-derived predictors and cohort-derived functional impairments and phenotypic frailty were associated with higher subsequent healthcare expenditures in community-dwelling beneficiaries.

**Objective:**

Determine calibration and discrimination of published cost prediction models among a nationally representative sample of US community-dwelling older adults in an independent sample (external validation) and at a different time-point (temporal validation).

**Design:**

Prospective cohort study

**Setting:**

National Healthy Aging and Trends Study (NHATS) 2015 cohort

**Participants:**

3,322 community-dwelling NHATS participants enrolled in Medicare fee-for-service.

**Measurements:**

Self-reported functional impairments (difficulty performing four activities of daily living) and frailty phenotype (operationalized using five components) derived from cohort data. Weighted (CMS Hierarchical Conditions Categories index) and unweighted (count of conditions) multimorbidity measures and frailty index derived from claims. Annualized healthcare costs ascertained for 36 months following 2015 examination. Cost ratios estimated from generalized linear models stratified by sex and adjusted for geographic region. Model calibration (predicted vs. observed costs by quintile) and discrimination (area under curve [AUC]) assessed.

**Results:**

In external and temporal validation cohorts, calibration was acceptable for all models with substantial agreement between predicted and observed costs. In the external cohort, the full model including claims- and cohort-derived variables had AUC = 0.78, 95% CI (0.74–0.82) in women and AUC = 0.77, 95% CI (0.73–0.81) in men, slightly higher than models limited to only claims-derived variables. Similar findings were noted in the temporal validation cohort.

**Conclusions:**

Performance of a model predicting total healthcare costs including self-reported functional impairments and phenotypic frailty together with claims-derived cost indicators is similar in a new independent cohort and in a subset of the original cohort at a later time-point. These results support clinical assessment of these geriatric syndromes for predicting risk of subsequent healthcare burden.

## Introduction

Our previous work sought to better identify older adults at risk for costly care to improve targeting of interventions to reduce overall healthcare burden. The starting point for such assessments is often administrative claims data, e.g., Medicare claims. The Center for Medicare & Medicaid Services Hierarchical Conditions Categories (CMS-HCC) model is commonly used as a weighted multimorbidity measure in cost prediction models in Medicare beneficiaries.[1,2] Likewise, claims-based frailty indices (CFI) might be used to capture deficit accumulation that may also lead to higher subsequent costs and provide additional predictive value.[3–5] Consistent with previous studies, we found that the CMS-HCC score, CFI, and a claims-based count of medical conditions were each independent predictors of subsequent healthcare costs in a model including only claims-based measures.[6]

While claims-based measures predict subsequent healthcare outcomes including total healthcare costs, they are derived from data created for payments and therefore may be missing other key cost indicators. Phenotypic frailty [7] assessed from clinical measures has been found to be associated with subsequent costs in many studies.[8–14] Clinical functional status assessments [15] may also be useful for the identification of those at risk of high healthcare utilization.[16–19] We previously reported that phenotypic frailty and functional impairments derived from cohort data were associated with substantial additional healthcare costs after accounting for claims-based cost indicators.[6] Both the claims-derived model and the full model (claims-derived model plus frailty and functional impairments) were internally cross-validated; full models including the cohort-derived variables performed better than the models with claims-derived variables alone. While internal validation provides some support for model robustness by using models developed in one subsample and evaluated in another subsample, TRIPOD guidelines note that external validation is essential for the assessment of model performance.[20]

Our primary aim was to perform an external validation of the main models created from the original multi-cohort analysis to predict annualized costs for the subset of 1688 participants in the NHATS 2015 exam who were newly recruited to NHATS in 2015 (and therefore were an independent sample). Our secondary aim was to perform a temporal validation of the original models using 1634 active surviving 2011 NHATS participants who were previously included in the model development as a subset of the cross-cohort.

## Methods

### Study Population and Linkage to Inpatient Claims

**Fig 1:**
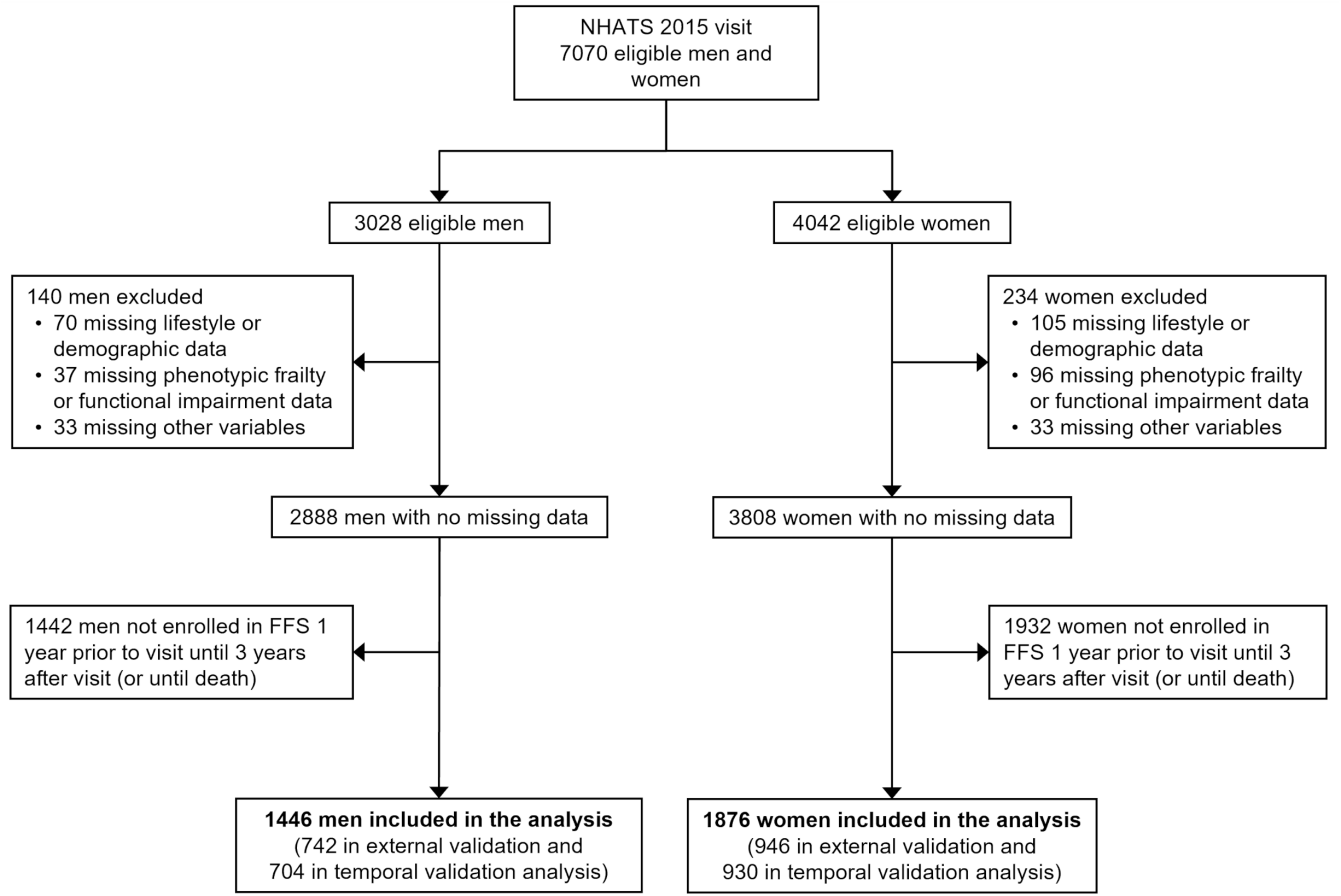
Participant Flow Diagram.

The eligible study population includes 3,322 community-dwelling older adults (Fig 1) enrolled in the 2015 examination of the National Health and Aging Trends Study (NHATS) with the requisite data for model validation. Those eligible had a comprehensive in-person examination including assessment of self-reported functional status (ability to perform basic and instrumental activities of daily living [ADL]) and the frailty phenotype and were also enrolled in the Medicare fee-for-service (FFS) program from 12 months prior to the index examination until 36 months following the index examination (or up until death within this period) (Fig 1). The eligible participants were divided into two mutually exclusive study populations. The main external validation cohort for the primary analysis consisted of 1,688 eligible NHATS participants (946 women, 742 men) who were newly recruited to participate in 2015. The temporal validation cohort included the 1,634 NHATS participants (730 women, 704 men) who were initially enrolled in 2011, but who also attended the 2015 NHATS clinic visit.

The study was approved by the University of Minnesota Human Research Protection Program IRB (FWA number 00000312). The IRB has issued a waiver of the consent process for the research question in this study. The analysis involved secondary analysis of cohort data linked with claims data. The IRB, serving as the HIPAA Privacy Board, issued a waiver of the requirement to obtain HIPAA Authorization to conduct research with this data.

Independent predictor variables were collected from study cohort data at the index examination as well as from claims data prior to the index visit. Cohort data from the index visit was used to assess functional impairments and the frailty phenotype. The period prior to the index exam in each cohort was used to calculate an unweighted claims-based measures of multimorbidity (i.e., count of chronic medical conditions), a weighted claims-based measure of multimorbidity (i.e., the CMS-HCC score), and a claims-based deficit accumulation frailty index (CFI).

### Independent Variables Derived from Cohort Data

Participants were asked at the index examination about difficulty performing four ADLs (i.e., walking a few blocks on level ground, climbing up 10–20 steps, transferring from bed to chair, and bathing or showering). Participants were classified as having none (referent group) up to four functional impairments. The frailty phenotype was assessed for each participant at the index examination using a modified version of five components initially proposed by Fried and colleagues [7] including shrinkage (recent weight loss of ≥5% or ≥10 pounds or BMI < 18.5 kg/m^2^), [9] weakness (grip strength <32 kg for men or <20 kg for women), [21] self-reported poor energy, slowness (gait speed <0.8 m/s for men or <0.6 m/s for women or use of a walking aid), [9,22,23] and low physical activity (reporting never walking for exercise and not engaging in moderate or vigorous activity) [24]. Participants were classified as robust (no components, referent group), having phenotypic pre-frailty (1 or 2 components) or having phenotypic frailty (3 or more components).

### Independent Variables Derived from Claims Data

A weighted claims-based multimorbidity measure, i.e., the Hierarchical Conditions Categories [CMS-HCC] model, was calculated for each participant using demographic data (age, sex, Medicaid eligibility, and disability status) and diagnosis codes from claims in the year prior to the index examination.[1,2] An unweighted multimorbidity measure (that is, count of conditions) was created by applying standard algorithms [25] to identify the presence or absence of each of 26 conditions selected by the CMS Chronic Conditions Data Warehouse (CCW) prior to index exam. The Kim claims-derived frailty indicator (CFI) approximating the deficit accumulation index [5] was calculated using diagnosis and procedure codes in the 12 months prior to the index examination.

### Dependent Variable Derived from Claims Data

Standardized annualized total direct healthcare costs for 36 months following the index examination was calculated and included costs paid by Medicare, costs paid by supplementary insurance, and out-of-pocket payments made by patients. Costs were annualized accounting for person-time and adjusted to U.S. 2023 dollars. Total direct annualized healthcare costs were calculated as the sum of costs for acute hospital stays, inpatient rehabilitation facility stays, skilled nursing facility stays paid at least in part under Medicare part A, outpatient care, durable medical equipment, and home healthcare for the 36-month time period.

### Statistical Analysis

The primary analyses (external validation) was performed using 1688 NHATS participants who were newly enrolled in 2015. A secondary analysis (temporal validation) was performed using the 1,634 NHATS participants who were initially enrolled in 2011, but who also attended the 2015 NHATS clinic visit. The models being validated were based on a development cohort that included NHATS participants who were enrolled in 2011 as well as participants from 3 other cohorts.[6] Thus, the secondary analysis is a further assessment of whether model coefficients are stable over time, but it does not have the study sample independence needed for external validation due to study sample overlap with the development cohort.

All cost models were stratified by sex and adjusted for geographic region as done in the development cohort.[10] The claims-based model included the CMS-HCC score, count of medical conditions, and Kim CFI as independent variables. The log-transformed HCC score and the Kim CFI score were standardized. The count of medical conditions was expressed as a categorical variable (0−1 condition [referent group], 2−4 conditions, ≥ 5 conditions). The full model contains both these claims-based measures of multimorbidity and frailty and the following cohort-derived variables: categorical frailty phenotype defined as robust [referent group], prefrail, or frail; and functional impairments defined as 0 [referent group], 1, 2, 3 or 4 impairments. The predictor variables are correlated (Kendall’s tau range 0.19–0.50). Measures from the same source (claims or cohort) were more correlated than variables from different sources. There was modest but acceptable variance inflation present in the original full model (maximum VIF = 3.69 for frailty in women). We updated the outcomes to 2023 costs for all models. We derived predicted costs for the NHATS 2015 cohort (external and temporal validation cohorts) using model parameters from the development cohort.

To assess calibration, mean predicted costs were compared to observed costs for risk subgroups based on quintiles of model predicted costs, category of the frailty phenotype, and number of functional impairments. To assess global discrimination, we used area under the receiver operator curve (AUC) with the binary outcome variable as having observed total costs in the top quartile. Our reference model for comparing discrimination AUC between the full model and claims-based models was the full model (including both claims-derived and cohort-derived variables).

To compare model parameters between different cohorts, we constructed new cost prediction models from NHATS 2015 data. The log-transformed HCC score and the Kim CFI score were standardized to match standardization values in the original development cohort. As done for the internal validation cohort, [6] the associations of predictor variables with annualized total direct healthcare costs over the subsequent 36 months were estimated using generalized linear models (GLMs) with log links and gamma distributions.

All analyses were performed using Stata version 18.0.

## Results

The combined analytical cohorts of eligible community-dwelling NHATS 2015 participants consisted of 1,876 women and 1,446 men. Of these, 946 women and 742 men were newly enrolled in 2015 (external validation cohort), while 930 women and 704 men were initially enrolled in 2011 (temporal validation cohort). Characteristics of both cohorts at the 2015 exam are shown in Table 1. The mean (SD) age in the external validation cohort was 76.8 (7.8) years in women and 75.7 (7.3) years in men, while women and men in the temporal validation cohort had mean age approximately 80 years. The median HCC score was 0.9 for both sexes in the external validation cohort, while it was 1.0 in the temporal validation cohort consistent with the higher expected values among the older cohort. The median Kim CFI score was higher in women than men in both the external and temporal validation cohorts; thus, women had slightly more accumulated deficits. In the external validation cohort, 498 (52.6%) women and 277 (37.3%) men reported at least 1 functional impairment, and 324 (34.2%) women and 195 (26.3%) men were classified as frail using the phenotypic definition. In the temporal validation cohort, 541 (58.2%) women and 276 (39.2%) men reported at least 1 functional impairment, and 334 (35.9%) women and 218 (31.0%) men were classified as frail using the phenotypic definition. During the 36 months following the 2015 examination, mean (SD) annualized costs in 2023 dollars were $16,054 ($25,047) for women and $17,491 ($28,930) for men in the external validation cohort and $17,685 ($ 27 030) for women and $19,315 ($33,905) for men in the temporal validation cohort.

**Table 1:** Selected Characteristics of Participants from 2015 National Healthy Aging and Trends Study (NHATS).

		External Validation Cohort^a^		Temporal Validation Cohort^b^	
		Women N = 946	Men N = 742	Women N = 930	Men N = 704
Age, yrs, mean (SD)		76.8 (7.8)	75.7 (7.3)	80.6 (6.9)	79.8 (6.5)
HCC score	mean (SD)	1.2 (1.1)	1.2 (1.2)	1.4 (1.1)	1.3 (1.1)
	median [IQR]	0.9 [0.5-1.4]	0.9 [0.5-1.5]	1.0 [0.7-1.7]	1.0 [0.7-1.7]
Kim claims-based frailty score	mean (SD)	0.18 (0.07)	0.16 (0.06)	0.18 (0.07)	0.16 (0.06)
	median [IQR]	0.16 [0.13-0.21]	0.15 [0.11-0.18]	0.17 [0.14-0.22]	0.15 [0.12-0.19]
Annualized total costs in US$	mean (SD)	16,054 (25,047)	17,491 (28,930)	17,685 (27 030)	19,315 (33,905)
	median [IQR]	7,085 [2,847−18,997]	7,190 [2,642−20,638]	7,909 [3,468−20,783]	8,580 [3,205−22,626]
Multimorbidity category, N (%)	0-1 condition	168 (17.8)	176 (23.7)	119 (12.8)	143 (20.3)
	2-4 conditions	395 (41.8)	287 (38.7)	396 (42.6)	263 (37.4)
	5 + conditions	383 (40.5)	279 (37.6)	415 (44.6)	298 (42.3)
Frailty Category, N (%)	Robust	175 (18.5)	152 (20.5)	143 (15.4)	142 (20.2)
	Pre-frail	447 (47.3)	395 (53.2)	453 (48.7)	344 (48.9)
	Frail	324 (34.2)	195 (26.3)	334 (35.9)	218 (31.0)
Functional impairments, N (%)	None	448 (47.4)	465 (62.7)	389 (41.8)	428 (60.8)
	1 impairment	150 (15.9)	107 (14.4)	171 (18.4)	91 (12.9)
	2 impairments	151 (16.0)	80 (10.8)	165 (17.7)	93 (13.2)
	3 impairments	98 (10.4)	42 (5.7)	114 (12.3)	37 (5.3)
	4 impairments	99 (10.5)	48 (6.5)	91 (9.8)	55 (7.8)
Death during 3-year follow-up, N (%)		104 (11.0)	103 (13.9)	155 (16.7)	139 (19.7)

a External validation cohort included newly recruited community-dwelling 2015 NHATS participants linked to Medicare data

bTemporal validation cohort included community-dwelling NHATS 2015 participants recruited in 2011 linked to Medicare data

### External and Temporal Validation

**Fig 2:**
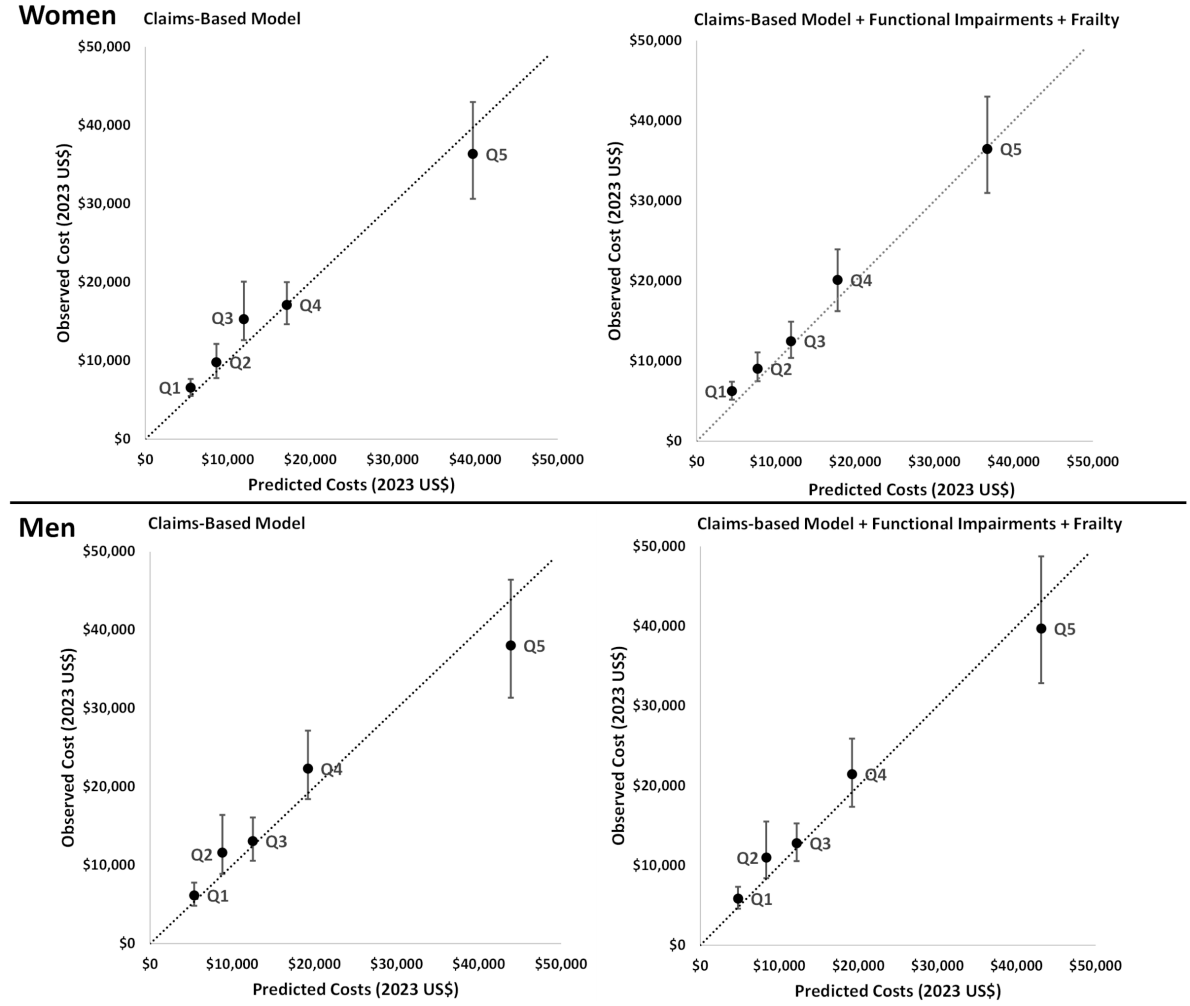
External Validation: Observed vs Predicted Healthcare Costs in Women and Men by Quintiles of Predicted Costs.

**Fig 3:**
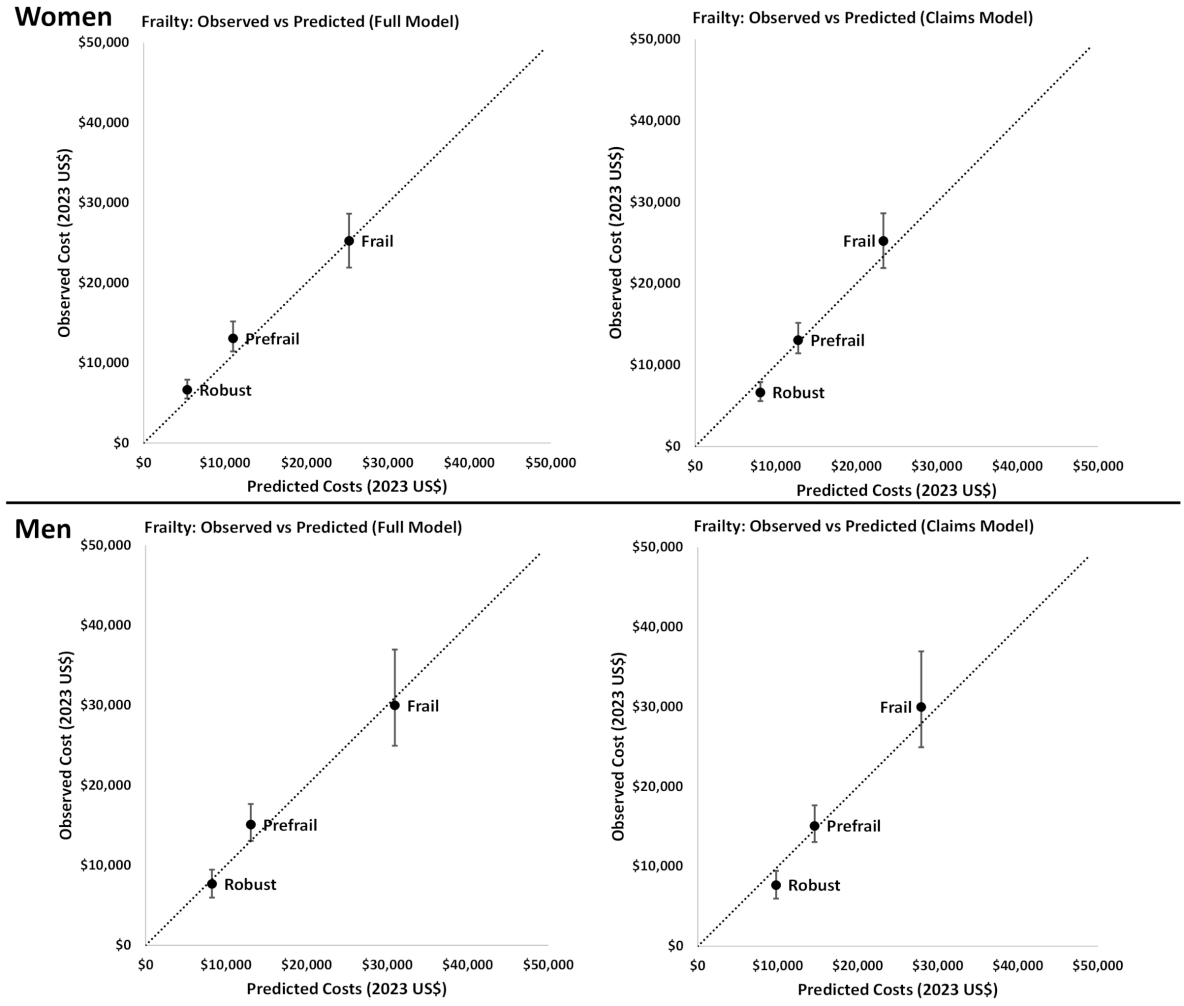
External Validation: Observed vs Predicted Healthcare Costs in Women and Men by Category of Frailty Phenotype (Robust, Prefrail, Frail).

**Fig 4:**
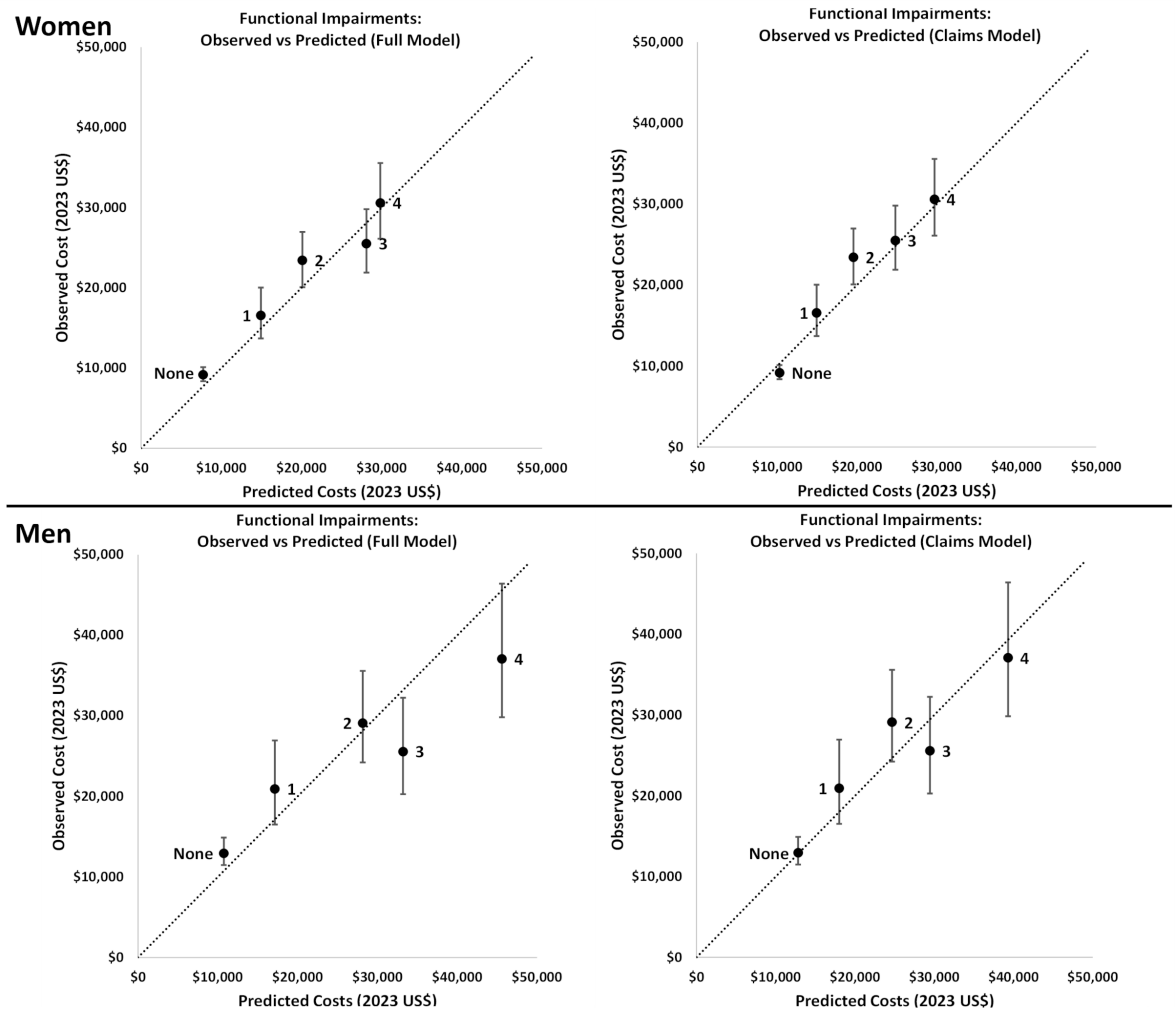
External Validation: Observed vs Predicted Healthcare Costs in Women and Men by Category of Functional Impairment (None, 1, 2, 3, or 4).

Calibration within quintiles of predicted costs in the external validation cohort was acceptable for models based on claims-derived variables alone and full models with substantial agreement between actual observed and predicted costs (Fig 2). In both the claims model and the full model, observed costs tended to be slightly higher than predicted costs in the lower quintiles for both men and women. Calibration within categories of frailty (Fig 3) and functional impairments (Fig 4) was acceptable for models based on claims-derived variables alone and full models with substantial agreement between actual observed and predicted costs. Similar findings were noted in the temporal validation cohort (S1 Fig, S2 Fig, S3 Fig).

Predicted costs based on the full model including both claims- and cohort-derived variables (Table 2) had good discrimination in the external validation cohort for high costs (top quartile of observed costs) in both sexes with AUC = 0.78, 95% CI (0.74–0.82) in women and AUC = 0.77, 95% CI (0.73–0.81) in men. Discrimination with the full model was notably better compared with the model discrimination of age alone and slightly better or at least as good compared with models based on HCC alone, CFI alone, or a claims-based model including HCC, CFI and count of medical conditions. Predicted costs based on the full model including claims and cohort variables (Table 2) also had good discrimination in the temporal validation cohort for high costs (top quartile of observed costs) in both sexes with AUC = 0.76, 95% CI (0.72–0.79) in women and AUC = 0.76, 95% CI (0.72–0.80) in men. Again, discrimination of the full model was notably better compared with the model discrimination of age alone and slightly better or at least as good compared with HCC alone, CFI alone, or a claims-based model including HCC, CFI and count of medical conditions.

**Table 2:** Model Discrimination for Prediction of High^a^ Annualized Total Healthcare Costs.

	External Validation Cohort^b^				Temporal Validation Cohort^c^			
	Women		Men		Women		Men	
Model	AUC^d^ (95% CI)	Comparison p-value	AUC^d^ (95% CI)	Comparison p-value	AUC^d^ (95% CI)	Comparison p-value	AUC^d^ (95% CI)	Comparison p-value
Full model^e^	0.78 (0.74 - 0.82)	Ref	0.77 (0.73 - 0.81)	Ref	0.76 (0.72 - 0.79)	Ref	0.76 (0.72 - 0.80)	Ref
Claims model^f^	0.77 (0.73 - 0.80)	0.05	0.76 (0.72 - 0.79)	0.04	0.75 (0.71 - 0.78)	0.35	0.75 (0.71 - 0.79)	0.06
Age alone	0.61 (0.57 - 0.66)	<0.001	0.64 (0.59 - 0.68)	<0.001	0.59 (0.55 - 0.63)	<0.001	0.61 (0.57 - 0.66)	<0.001
HCC score alone	0.75 (0.72 - 0.79	0.005	0.76 (0.72 - 0.80)	0.22	0.74 (0.71 - 0.78)	0.15	0.74 (0.70 - 0.78)	0.04
CFI score alone	0.76 (0.72 - 0.79)	0.07	0.70 (0.66 - 0.75)	<0.001	0.71 (0.67 - 0.75)	0.001	0.71 (0.67 - 0.75)	0.0005

a Top quartile of total healthcare costs in respective cohort

b External validation cohort included newly recruited community-dwelling 2015 NHATS participants linked to Medicare data

c Temporal validation cohort included community-dwelling NHATS 2015 participants recruited in 2011 linked to Medicare data

d Area under Receiver operator curve (AUC)

e Full model in development cohort included cohort-derived variables (frailty phenotype and functional impairments), claim-based variables (Hierarchical Conditions Categories (HCC) score, multimorbidity, Kim claims-based frailty index (CFI)) and was adjusted for geographic region

f Claims model in development cohort included Hierarchical Conditions Categories (HCC) score, multimorbidity, Kim claims-based frailty index (CFI) and was adjusted for geographic region

### Associations of Claim-Based Measures, Functional Impairments, and Phenotypic Frailty with Annualized Healthcare Costs

There was some slight inconsistency in model parameters between the development and external validation cohort (Table 3). For women, the HCC score predicted total costs in both the development and external cohorts, but the point estimates of the association were somewhat lower for the external validation [cost ratio = 1.28, 95% CI (1.16–1.43)] versus the development cohort [cost ratio = 1.43, 95% CI (1.32–1.55)]. For men, the HCC score also predicted total costs in both the development and external cohorts, but the point estimates of association were somewhat higher for the external validation [cost ratio = 1.67, 95% CI (1.48–1.90)] versus the development cohort [cost ratio = 1.49, 95% CI (1.37–1.62)]. For women, the Kim CFI score predicted costs in both the development and external validation cohorts, whereas for men the association was not statistically significant in either cohort. We note that the sample size in both external validation cohorts is smaller than the development cohort, therefore resulting in wider confidence intervals. For both sexes, there were associations between the number of medical conditions and costs such that the point estimates of the cost ratios for counts of 2–4 conditions and 5 + conditions compared with referent group of 0–1 condition were all higher than 1.00, but the associated confidence intervals around the point estimates in the external validation cohort were not consistently above 1.00. For both men and women, predicted costs were generally higher for those with a greater number of functional impairments in both cohorts. Again, in the external validation cohort, the associated confidence intervals around the point estimates of associations were not consistently above 1.00 nor was there a consistent monotonic increase. For women, frailty phenotype category (frail versus robust) predicted total costs in both the development and external validation cohorts, but the point estimates of association were somewhat lower for the external validation [cost ratio = 1.38, 95% CI (1.02–1.87)] versus the development cohort [cost ratio = 1.71, 95% CI (1.40–2.08)]. For men, phenotypic frailty was not an independent predictor of costs in the development cohort, but there was an association in the external validation cohort [cost ratio = 1.50, 95% CI (1.04–2.17)].

**Table 3:** Comparisons of Model Parameters^a^ for Prediction of Annualized Total Healthcare Costs Using Different Cohorts.

		Cost Ratios (95% CI)					
		Women			Men		
Parameter		Ensrud et al multi-study cohort^b^	NHATS external validation cohort^c^	NHATS temporal validation cohort^d^	Ensrud et al multi-study cohort^b^	NHATS external validation cohort^c^	NHATS temporal validation cohort^d^
Log HCC (per SD)		1.43 (1.32 - 1.55)	1.28 (1.16 - 1.43)	1.52 (1.35 - 1.72)	1.49 (1.37 - 1.62)	1.67 (1.48 - 1.90)	1.44 (1.23 - 1.69)
CFI score (per SD)		1.11 (1.02 - 1.20)	1.16 (1.04 - 1.28)	1.08 (0.96 - 1.20)	1.07 (0.98 - 1.17)	0.89 (0.78 - 1.01)	1.00 (0.85 - 1.17)
Multimorbidity	0-1 condition	Ref	Ref	Ref	Ref	Ref	Ref
	2-4 conditions	1.22 (1.04 - 1.42)	1.08 (0.85 - 1.37)	1.10 (0.84 - 1.44)	1.39 (1.20 - 1.61)	1.19 (0.91 - 1.57)	1.35 (0.97 - 1.87)
	5 + conditions	1.18 (0.97 - 1.43)	1.22 (0.91 - 1.64)	1.15 (0.84 - 1.57)	1.48 (1.22 - 1.81)	1.17 (0.83 - 1.64)	1.72 (1.15 2.59)
# of functional impairments	None	Ref	Ref	Ref	Ref	Ref	Ref
	1	1.24 (1.06 - 1.44)	1.26 (0.97 - 1.63)	1.16 (0.90 - 1.49)	1.17 (0.99 - 1.39)	1.08 (0.80 - 1.47)	1.17 (0.81 - 1.70)
	2	1.33 (1.13 - 1.58)	1.52 (1.16 - 2.00)	1.55 (1.18 - 2.02)	1.45 (1.17 - 1.80)	1.38 (0.96 - 1.97)	1.28 (0.87 - 1.88)
	3	1.54 (1.24 - 1.92)	1.37 (0.98 - 1.91)	1.68 (1.23 - 2.30)	1.53 (1.16 - 2.03)	1.40 (0.88 - 2.23)	0.83 (0.47 - 1.48)
	4	1.39 (1.08 - 1.80)	1.60 (1.14 - 2.25)	1.46 (1.01 - 2.11)	1.79 (1.31 - 2.45)	1.63 (0.99 - 2.68)	1.55 (0.95 - 2.55)
Frailty category	Robust	Ref	Ref	Ref	Ref	Ref	Ref
	Prefrail	1.32 (1.12 - 1.55)	1.22 (0.96 - 1.56)	1.17 (0.91 - 1.51)	1.02 (0.89 - 1.17)	1.38 (1.06 - 1.81)	1.25 (0.91 - 1.71)
	Frail	1.71 (1.40 - 2.08)	1.38 (1.02 - 1.87)	1.10 (0.80 - 1.52)	1.20 (0.98 - 1.48)	1.50 (1.04 - 2.17)	1.54 (1.04 - 2.28)

a Cost Ratios = exponentiated model coefficients

b Cost ratios from development cohort: Multi-study cohort included Study of Osteoporotic Fractures (SOF), Osteoporotic Fracture in Men Study (MrOS), Health, Aging and Body Composition Study (HABC), and National Health and Aging Trends Study (NHATS 2011)

c Cost ratios from community-dwelling NHATS participants recruited in 2015 (external validation cohort)

d Cost ratios from community-dwelling NHATS 2015 participants recruited in 2011 (temporal validation cohort)

There was also some inconsistency in model parameters between the development and temporal validation cohort (Table 3). The overall direction of differences varied, but the confidence intervals still substantially overlapped (e.g., point estimate of temporal validations cohort was within CI of development cohort) with a few exceptions. For women, frailty phenotype category (frail versus robust) was not associated with costs in the temporal validation cohort [cost ratio = 1.10, 95% CI (0.80–1.52)] while the association was strong and significant in the development cohort [cost ratio = 1.71, 95% CI (1.40–2.08)]. The converse was true for men, so that frailty phenotype category (frail versus robust) was associated with costs in the temporal validation cohort [cost ratio = 1.54, 95% CI (1.04–2.28)] while it was not significant in the development cohort [cost ratio = 1.20, 95% CI (0.98–1.48)].

## Discussion

We assessed the calibration and discrimination of our previously published models using claims-based variables alone and a combination of claims and cohort-derived variables for the prediction of total healthcare costs.[6] Our results indicate that the original models had acceptable performance both in the primary independent external validation cohort and in the secondary temporal validation cohort. The calibration of all models was acceptable with no clear differences between the claims-based model and the full models. The discrimination of the full model was slightly better than that of the claims-based model. There were no notable differences in model performance for the external and temporal validation cohorts. Thus, while there was some study sample overlap between the development cohort and the temporal validation cohort, this overlap did not seem to impact the overall findings about model performance. Small deviations in calibration, i.e., calibration slope less than one, were noted in both external and temporal validation analyses. Standard linear regression models will on average appear better in the development cohort than in the validation cohort, i.e., there is a tendency for the model to overfit the data. One result of overfitting is that predictor variable coefficients are biased away from the null. Overfitting will appear in external validation as model shrinkage, i.e., the calibration slope will be less than one. Discrimination as determined by AUC was within the acceptable range for both the external and temporal validation cohorts. We also compared model parameters for cohort measures in the 3 different cohorts. There was some variation in the point estimates and null hypothesis tests between the three cohorts, but associations of predictor variables with total costs were largely consistent across cohorts. In summary, our findings support the validity of a cost prediction model in community-dwelling adults including a combination of both cohort-based and claims-derived independent variables.

The current study provides support that phenotypic frailty is associated with excess costs in community-dwelling older adults after accounting for comprehensive claims-based measures of multimorbidity and self-reported functional impairments. This finding extends previous results reported for single cohort studies [9,10] as well as in the multi-cohort study of Ensrud et al [6] which included participants from the two earlier single cohort studies. Chi et al [26] performed a meta-analysis of 7 studies and found that both phenotypic frailty status and its individual components were associated with increased healthcare costs. The meta-analysis provided further support for our initial papers, but of note it included the two earlier papers [9,10] from our research group. There are other studies with independent confirmatory results reporting that older adults with phenotypic frailty compared with individuals who are robust have consistently higher healthcare costs.[8,11–14] There are some discrepancies in the association between frailty and healthcare costs, notably the attenuation after further adjustment for functional impairments in men in the multi-cohort study.[6] Because frailty and functional impairments commonly co-occur, this discrepancy may be related to more unstable parameter estimates due to the substantial overlap between these 2 geriatric syndromes.

The current study also provides additional evidence that functional impairments are associated with increased costs after adjustment for comprehensive claims-based measures of multimorbidity and the frailty phenotype. Our work on functional impairments was performed concurrently with assessments of frailty with both single cohort [9,10] and the multi-cohort study.[6] The two initial studies focused on frailty per se and adjusted for functional impairments, whereas the latter study treated frailty phenotype and functional impairments as two primary predictors of interest. There was a clear graded response between a greater number of functional impairments and higher costs in men for the multi-cohort study and the present study, while a weaker trend was noted for women in both studies. Relating these results to other studies is challenging. Many prior studies [16–19] focused on risk assessment adjusted for multimorbidity, while the full model in the present study adjusted for a comprehensive set of variables including cohort-based and claims-derived variables associated with frailty. A recent review presented ample evidence that functional impairments considered in a broader sense (incorporating frailty, physical performance, chronic conditions, cognitive impairment, depression) are associated with higher total healthcare costs.[27] Our results on functional impairments fit into the more general context reported in the paper, but separate out costs attributable to various combinations of phenotypic frailty and functional impairments.

The HCC model is based on principles designed to capture a specific and clinically meaningful number and complexity of medical conditions related to future healthcare burden as measured by costs.[2] We have used the HCC model as a gold standard from which to assess cost prediction. More recent work of Andriola et al [28] has shown that machine learning methods leveraging large data from the Merative MarketScan Commercial Claims and Encounters Database can improve healthcare cost prediction compared with the HCC model, particularly for those with rare diseases. In this study, the final model improved cost prediction, but was based on a more extensive hierarchical risk set with over 600 model parameters. In contrast, our claims model includes a parsimonious set of predictors (HCC score, Kim CFI score, and count of chronic medical conditions), but still slightly improved discrimination of high costs compared with a model based on HCC score alone. The full model further improved discrimination, but again with only a minimal increase in complexity compared to the claims model. We note, however, that claims variables by their nature do not require resources or documentation beyond that already performed during clinical visits to compute the measure. In contrast, the proposed clinical measures require time, personnel, space, and documentation for each visit at which they are assessed in resource strained clinical settings. Thus, while the clinical measures are simple, they have costs associated with implementation. Further research should elucidate whether assessment of the frailty phenotype and functional impairments enhances clinical decision making given these considerations. Mehta et al [29] performed a comparative analysis of comorbidity scores for the prediction of adverse outcomes following surgery and found that a surgery‐specific comorbidity score performed better than other scores (including HCC score) for length of stay and mortality outcomes following surgery. General purpose models such as the HCC will likely yield acceptable performance in many contexts, but other models might be better when derived for a given population and outcome.

### Strengths and limitations

We have now assessed models in a large multi-cohort study with internal validation and a second independent sample in an external validation study, thus showing predictions may be used outside the cohort where they were defined. Using models including both cohort-based and claims-derived independent variables demonstrates potential limitations of relying on models using claims-based measures alone, particularly for constructs that require further assessment or do not have specific diagnosis codes (e.g., phenotypic frailty and self-reported functional impairments). One of the limitations of this approach is generalizability, since the model was developed using fee-for-service Medicare claims in the United States linked with cohort data and therefore estimated parameters are specific to similar data in similar context (i.e., those enrolled in fee-for-service Medicare). CMS allows linkage for research purposes, but such linkage is limited by strict data use agreements and availability of cohort data. The HCC score in particular is developed for risk adjustment based on US Medicare claims, but others have applied a version of HCC to South Korean claims data [30]. This application suggests possible replication of the conceptual models in other data sets. Both the claims model and the full model included independent variables that were correlated. This choice results in higher uncertainty (i.e., wider confidence intervals) of the model parameters due to variance inflation. Any interpretation of the model parameters should consider this uncertainty. We acknowledge the limited sample size of both the external validation cohort and the temporal validation cohort. Trends resulting in overall changes in calibration are still possible. The small sample size also resulted in model parameter uncertainty with large confidence intervals, particularly notable for categorical variables. An additional limitation of our analysis is that while the models appear stable over time and we have updated costs to account for inflation, the models are still based on older data. Secular changes, particularly in regard to the target population (enrollees in FFS Medicare) may have changed over time.

Those who died prior to 36 months were included in the analysis but with the observed person-time until death used as the scale factor. This may result in exceptionally annualized costs, particularly for those with total observation period of less than one year where the annualization is effectively an extrapolation from a shorter intense health care period to a longer one. For those with longer periods of observation, annualized costs reflect true average rates accounting for variable time of observation. The underlying question is whether and how early mortality should be accounted for and ranked in the analysis. Conceptually, we thought of total health care costs as a proxy for overall burden of disease. Thus, the fact that early death might inflate costs when annualized is consistent with the idea that early mortality reflects higher burden.

## Conclusion

In summary, we found that a cost prediction model combining clinical measures derived from cohort data and measures derived from claims data had acceptable performance in external and temporal validation cohorts. As the source data for the development cohort spanned the years 2002–2011, our validation in the 2015 NHATs cohort indicates that there was no marked degradation in model performance over time. Small deviations in calibration consistent with model shrinkage were noted, but these deviations are in general expected. Overall model discrimination was good in the external validation sample. Future costs are related to future and unknowable events, and this is reflected by upper bounds on the ability of any model to predict high costs. Results of our validation analyses confirm that models using claims-derived variables alone predict total healthcare costs in community-dwelling adults, but our findings also suggest that other measures not typically captured in claims data such as the frailty phenotype and self-reported functional impairments are relevant as independent predictors of costs as their assessment may improve characterization of individuals at increased risk of costly care.

## Supporting information

S1 Fig Temporal Validation: Observed vs Predicted Healthcare Costs in Women and Men by Quintiles of Predicted Costs. (DOCX)

S2 Fig Temporal Validation: Observed vs Predicted Healthcare Costs in Women and Men by Category of Frailty Phenotype (Robust, Prefrail, Frail). (DOCX)

S3 Fig Temporal Validation: Observed vs Predicted Healthcare Costs in Women and Men by Category of Functional Impairment (None, 1, 2, 3, or 4). (DOCX)
