# Supplementary material for: External validation of model predicting healthcare costs using cohort study-derived and claims-derived predictor variables in community-dwelling older adults
Source: PLoS One. 2026 Aug 12;21(8):e0354978. doi: 10.1371/journal.pone.0354978 (PMC13465825; doi:10.1371/journal.pone.0354978)
Supplement: Supplementary file 1 [file pone.0354978.s001.docx]

**Supplemental Fig 1. Temporal Validation: Observed vs Predicted Healthcare Costs in Women and Men by Quintiles of Predicted Costs.**
