# Supplementary material for: External validation of model predicting healthcare costs using cohort study-derived and claims-derived predictor variables in community-dwelling older adults
Source: PLoS One. 2026 Aug 12;21(8):e0354978. doi: 10.1371/journal.pone.0354978 (PMC13465825; doi:10.1371/journal.pone.0354978)
Supplement: Supplementary file 3 [file pone.0354978.s003.docx]

**Supplemental Fig 3. Temporal Validation: Observed vs Predicted Healthcare Costs in Women and Men by Category of Functional Impairment (None, 1, 2, 3, or 4).**
